# Psychedelic N,N-Dimethyltryptamine and 5-Methoxy-N,N-Dimethyltryptamine Modulate Innate and Adaptive Inflammatory Responses through the Sigma-1 Receptor of Human Monocyte-Derived Dendritic Cells

**DOI:** 10.1371/journal.pone.0106533

**Published:** 2014-08-29

**Authors:** Attila Szabo, Attila Kovacs, Ede Frecska, Eva Rajnavolgyi

**Affiliations:** 1 Department of Immunology, Faculty of Medicine, University of Debrecen, Debrecen, Hungary; 2 Research Centre for Molecular Medicine, University of Debrecen, Debrecen, Hungary; 3 Department of Psychiatry, Faculty of Medicine, University of Debrecen, Debrecen, Hungary; University of Cologne, Germany

## Abstract

The orphan receptor sigma-1 (sigmar-1) is a transmembrane chaperone protein expressed in both the central nervous system and in immune cells. It has been shown to regulate neuronal differentiation and cell survival, and mediates anti-inflammatory responses and immunosuppression in murine *in* *vivo* models. Since the details of these findings have not been elucidated so far, we studied the effects of the endogenous sigmar-1 ligands N,N-dimethyltryptamine (NN-DMT), its derivative 5-methoxy-N,N-dimethyltryptamine (5-MeO-DMT) and the synthetic high affinity sigmar-1 agonist PRE-084 hydrochloride on human primary monocyte-derived dendritic cell (moDCs) activation provoked by LPS, polyI:C or pathogen-derived stimuli to induce inflammatory responses. Co-treatment of moDC with these activators and sigma-1 receptor ligands inhibited the production of pro-inflammatory cytokines IL-1β, IL-6, TNFα and the chemokine IL-8, while increased the secretion of the anti-inflammatory cytokine IL-10. The T-cell activating capacity of moDCs was also inhibited, and dimethyltryptamines used in combination with *E. coli* or influenza virus as stimulators decreased the differentiation of moDC-induced Th1 and Th17 inflammatory effector T-cells in a sigmar-1 specific manner as confirmed by gene silencing. Here we demonstrate for the first time the immunomodulatory potential of NN-DMT and 5-MeO-DMT on human moDC functions via sigmar-1 that could be harnessed for the pharmacological treatment of autoimmune diseases and chronic inflammatory conditions of the CNS or peripheral tissues. Our findings also point out a new biological role for dimethyltryptamines, which may act as systemic endogenous regulators of inflammation and immune homeostasis through the sigma-1 receptor.

## Introduction

The term sigma receptor dates back historically to the sigma/opioid receptor described by Martin et al. [1] and reported to mediate the psychotropic effects of N-allylnormetazocine (NANM). It was originally thought to be an opioid receptor due to its modulation by NANM that could be antagonized by naloxone, a universal opioid antagonist [2]. Later, Su and colleagues clarified the pharmacological features of the ligand-binding site and the name was changed to ‘sigma receptor’ differentiating it from the sigma/opioid receptor [3], [4]. According to its tissue expression profile and ligand selectivity the receptor was subsequently classified to the sigma-1 and sigma-2 receptor subtypes (sigmar-1/2) [5]. In the last two decades several clinical studies demonstrated the importance of sigmar-1 in many diseases ranging from cancer, pain and addiction to different psychiatric and neurological disorders among them Major depression, Alzheimer’s disease, schizophrenia, and stroke [2].

Early studies showed that sigmar-1 is expressed not only in distinct regions of the CNS but also in immune cells [4], [6]. It was shown to regulate cell differentiation and survival by acting as a chaperone at the mitochondria-associated endoplasmic reticulum membrane [7], [8]. Murine studies also demonstrated that the specific activation of sigmar-1 resulted in immunosuppression [9], and *in* *vivo* decreased lymphocyte activation and proliferation [10]. Sigma-1 receptor ligands possess potent immunoregulatory properties via increasing the secretion level of anti-inflammatory IL-10 [11], and suppressing IFNγ and GM-CSF expression [10]. These important studies showed that sigmar-1 may cause significant alterations in immune functions.

The endogenous ligands for sigmar-1 involve neurosteroids, dehydroepiandrosterone (DHEA), and naturally occuring indole alkaloids/tryptamines, such as N,N-dimethyltryptamine (NN-DMT) and its closely related analogue 5-methoxy-N,N-dimethyltryptamine (5-MeO-DMT). Hallucinogen indole alkaloids are widespread in nature and abundant in plants, which are used in preparation of sacramental psychoactive decoctions such as *yage* and *ayahuasca* [12]. NN-DMT and 5-MeO-DMT have also been detected in animal tissues; furthermore, NN-DMT is considered as an endogenous trace amine neurotransmitter that regulates brain physiology [13]–[15]. It has recently been shown that NN-DMT is a natural ligand for sigmar-1 [16], and its administration was reported to influence the number of circulating lymphocytes in humans, but the exact mechanism has not been uncovered yet [17]. In the light of these findings it is tempting to speculate that NN-DMT and 5-MeO-DMT may have impact on inflammatory responses through sigmar-1 [12].

Dendritic cells (DCs) are key players of innate immunity in higher vertebrates and their most prominent functions involve the continous sampling of the neighbouring enviroment. Harboring a selected spectrum of pathogen-sensing pattern recognition receptors (PRRs), such as intracellular Toll-like receptors (TLRs) and RIG-I-like receptors (RLRs), they can be activated by specific self and non self ligands [18]. They also exhibit the unique and crucial ability to translate PRR-mediated signals to adaptive immunity, thereby orchestrating natural and acquired immune responses [19]. Specialized subsets of DCs are working together as a network of vigilant gatekeepers in literally all tissues of the body [20]. *In vitro* differentiated human monocyte-derived DCs (moDCs) are considered as gold standards of DC biology and are used in various clinical and experimental settings [21]. Since human monocytes have recently been shown to migrate to the brain and are able to modulate the neuroinflammatory profile of the CNS [22], moDCs may represent a cell type, which, besides microglia, could also contribute to the immunoregulation of the neural tissue.

In this study we aimed to investigate the effects of NN-DMT and 5-MeO-DMT-mediated activation of sigmar-1 on human primary moDC functions under inflammatory conditions as compared to resting state. To our best knowledge this is the first study reporting that dimethyltryptamines are potent anti-inflammatory agents, which have the capacity to modulate the functions of moDCs in a sigmar-1-dependent manner. Our results envision that dimethyltryptamines targeted to the sigmar-1 receptor could emerge as promising candidates for future pharmacological therapies in chronic inflammatory and autoimmune conditions of the CNS or peripheral tissues. We also propose a new biological role for NN-DMT, which, through the sigmar-1 of myeloid immune cells, may act as an endogenous regulator of inflammation and immune homeostasis.

## Materials and Methods

### Cell isolation and culturing

Leukocyte-enriched buffy coats were obtained from healthy blood donors drawn at the Regional Blood Center of the Hungarian National Blood Transfusion Service (Debrecen, Hungary) in accordance with the written approval of the Director of the National Blood Transfusion Service and the Regional and Institutional Ethics Committee of the University of Debrecen, Faculty of Medicine (Debrecen, Hungary). Written informed consent was obtained from the donors prior blood donation, and their data were processed and stored according to the directives of the European Union. Peripheral blood mononuclear cells (PBMCs) were separated by a standard density gradient centrifugation with Ficoll-Paque Plus (Amersham Biosciences, Uppsala, Sweden). Monocytes were purified from PBMCs by positive selection using immunomagnetic cell separation with anti-CD14 microbeads according to the manufacturer’s instruction (Miltenyi Biotech, Bergisch Gladbach, Germany). After separation on a VarioMACS magnet, 96–99% of the cells were CD14^+^ monocytes as measured by flow cytometry. Monocytes were cultured in 12-well tissue culture plates at a density of 2×10^6^ cells/ml in serum-free AIMV medium (Invitrogen, Carlsbad, CA) supplemented with 80 ng/ml GM-CSF (Gentaur Molecular Products, Brussels, Belgium) and 100 ng/ml IL-4 (Peprotech EC, London, U.K.). On day 2, the same amounts of GM-CSF and IL-4 were added to the cell cultures without changing their media.

Autologous naive T-cells were separated from mononuclear cells of the same donor using human naive CD4^+^ T Cell Isolation Kits (Miltenyi Biotech).

### Activation of dendritic cells

Bacterial lipopolysaccharide (LPS) (Sigma, Schnelldorf, Germany), and high molecular weight polyinosinic: polycytidylic acid (polyI:C) (InvivoGen, San Diego, CA) were used at working concentrations of 500 ng/ml (LPS) and 20 µg/ml (polyI:C), respectively. N,N-dimethyltryptamine (R&D Systems, Abingdon, UK), 5-methoxy-N,N-dimethyltryptamine (Sigma), and 2-(4-morpholinoethyl-1)-phenylcyclohexane-1-carboxylate hydrochloride (PRE084) (Tocris Bioscience, Bristol, UK) were used in a working concentration of 100 µM. Pre-treatments were performed 1 h prior to DC activation. Controlled substances (Schedule I drugs) were used with the approval and monitoring of the Hungarian Institute for Forensic Sciences and the Hungarian National Police Department.

Purified and inactivated A/Brisbane/59/7 (H1N1) influenza virus (kindly provided by the National Center for Epidemiology, Hungary) of 6×10^6^ PFU/mL was used for in vitro treatment of 1×10^6^ per mL DC in serum-free AIMV medium for 24 h. Activated cells were washed two times in sterile medium and then co-cultured with autologous naive T cells (ELISPOT).

For activation of cells with bacteria, 5×10^4^ heat-killed *Escherichia coli 058* (Institute of Microbiology and Virology of National Academy of Science, Hungary) was added to 10^5^ moDCs in 300 µl medium on a 96 well plate and incubated at 37°C for 24 h prior to ELISPOT assay. MoDCs were washed two times in sterile AIMV before co-culturing, as above.

To prepare cell lysates for Western blotting, or collect supernatants for ELISA cells were activated for 24 h. Cell lysates were made after 8 h for Q-PCR measurements (if not stated otherwise).

### RNA isolation, cDNA synthesis and QPCR

Real-time quantitative polymerase chain reaction (QPCR) was performed as described previously [23]. Briefly, total RNA was isolated by TRIzol reagent (Invitrogen, Carlsbad, CA). 1.5–2 µg of total RNA were reverse transcribed using SuperScript II RNase H reverse transcriptase (Invitrogen) and Oligo (dT) 15 primers (Promega, Madison, WI). Gene-specific TaqMan assays (Applied Biosystems, Foster City, CA) were used to perform QPCR in a final volume of 12 µl in triplicates using AmpliTaq Gold DNA polymerase and ABI StepOnePlus real-time PCR instrument (Applied Biosystems). Amplification of 36B4 was used as a normalizing control. Cycle threshold values (Ct) were determined by using the StepOne 2.1 software. Constant threshold values were set for each gene throughout the study. Details of TaqMan assays are shown in Table S1.

### Cytokine measurements

Culture supernatants were harvested 24 hours after activation and the concentrations of IL-1β, IL-6, TNFα, IL-8, and IL-10 cytokines were measured using OptEIA kits (BD Biosciences, San Jose, CA) following the manufacturer’s recommendations. The precision of the kits were the following: Intra-Assay variation: CV<10%; Inter-Assay variation: CV<12% (CV% = SD/meanX100). In each cases non-diluted supernatant samples were used for the assays, except IL-6 (3X dilution) and IL-8 (6X dilution) measurements.

### Western blotting

Cells were lysed in Laemmli buffer and the protein extracts were tested by Ab specific for OPRS1/Sigmar-1 (Abcam, Cambridge, UK), and β-actin (Sigma) diluted at 1∶500 and 1∶000, respectively. Anti-rabbit Ab conjugated to horseradish peroxidase (GE Healthcare, Little Chalfont Buckinghamshire, UK) was used as the secondary Ab at a dilution of 1∶5000. The SuperSignal enhanced chemiluminescence system was used for probing target proteins (Thermo Scientific, Rockford, IL). After the membranes had been probed for OPRS1/Sigmar-1, they were stripped and re-probed for β-actin.

### ELISPOT assay

Activated, pathogen-loaded DCs (2×10^5^ cells/well) were co-cultured with naïve autologous CD4^+^ T cells (10^6^ cells/well) in serum-free AIMV medium for 4 days at 37°C in humidified atmosphere containing 5% CO_2_. Phytohaemagglutinin (PHA) and Concanavalin A (ConA) activated T cells were used as positive controls, non-treated DC+T cell co-cultures and T cells without DC served as negative controls. Detection of activated, cytokine secreting CD4^+^ T cells producing IFNγ or IL17 was performed by the avidin-horseradish peroxidase system (NatuTec). Plates were analyzed on ImmunoScan plate reader (CTL Ltd., Shaker Heights, OH).

### RNA interference

Gene-specific siRNA knockdown was performed by Silencer Select siRNA (Applied Biosystems) transfection using Gene Pulser Xcell instrument (Bio-Rad, Hercules, CA). Pulse conditions were square-wave pulse, 500 V, 0.5 ms. Immediately after electroporation, cells were transferred to fresh AIMV medium supplemented with, penicillin, streptomycin, and L-glutamine in addition to GM-CSF and IL-4. Silencing of *sigmar1* gene expression was performed by using a mix of three of the available SIGMAR1 siRNAs. Silencer negative control nontargeting siRNA (Applied Biosystems) was used as a negative control. The efficacy of siRNA treatments was tested two days post-transfection by Western blotting.

### Statistical analysis

Data are presented as mean ± SEM. A *t-test* was used for comparison of two groups. One-way ANOVA, followed by Bonferroni post hoc test, was used for multiple comparisons. Differences were considered to be statistically significant at p values <0.05 (*).

## Results

### Expression of sigmar-1 in resting and activated human primary myeliod cells

**Figure 1:**
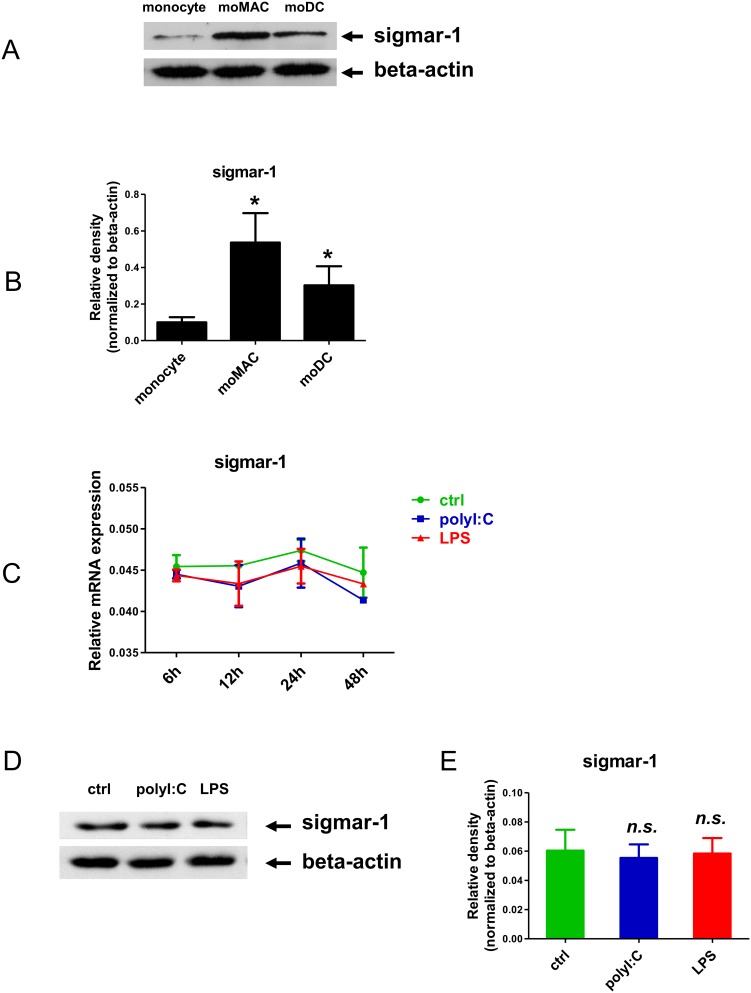
Expression of the Sigma-1 receptor in human monocytes, and differentiated monocyte-derived macrophages (moMACs) and dendritic cells (moDCs). **A–B**: Expression of sigmar-1 protein in monocytes, and differentiated moMACs and moDCs measured by Western blot. In Fig1A a typical experiment out of four is demonstrated. Densitometry data of Fig1B show the Mean ± SEM values of four independent donors. C: Time kinetics of sigmar-1 gene expression in non-activated control (ctrl), 20 µg/ml polyI:C (polyI:C) or 500 ng/ml LPS (LPS) treated cells. Data of triplicates of four independent measurements are show as Mean ± SEM. D–E: Sigmar-1 protein expression in non-activated control, 20 µg/ml polyI:C or 500 ng/ml LPS treated cells following 24 hours of activation. In Fig1D, results of a typical experiment out of four is shown. Densitometry data of Fig1E show the Mean ± SEM values of four independent donors. (*) represents *p* values<0.05. (n.s.: “non-significant”).

Sigmar-1 is an intracellular receptor that is located on the mitochondria-associated endoplasmic reticulum membrane [7]. It has already been detected in various neuronal cell types and brain macrophages (microglia) of rodents [24], [25], but little is known about its expression in other immune cells. We first sought to monitor the expression of sigmar-1 in human primary myeloid cells (Figure 1) and found that sigmar-1 was expressed in primary human blood monocytes and its expression increased during the differentiation process toward macrophages and moDCs shown by the increasing levels of sigmar-1 protein in both monocyte-derived macrophages (moMACs) and moDCs (Figure 1A and 1B). Interestingly, we could not detect measurable levels of sigmar-2 mRNA or protein in these samples derived from the same donors (data not shown). Similar to previous results obtained with murine microglia we also detected high levels of sigmar-1 protein in human moMACs [24], [25]. Since the expression and function of sigmar-1 has already been established in macrophages, and moDCs showed comparable levels of sigmar-1 to moMACs, in our further studies we focused on moDCs. Taken the versatile immunomodulatory potential of this regulatory cell type we first addressed the question whether inflammatory conditions could alter the expression of sigmar-1 in moDCs. To mimic inflammation and infection by pathogenic signals we treated moDCs with high-dose bacterial lipopolysaccharide (LPS) or the viral nucleic-acid analogue polyinosinic: polycytidylic acid (polyI:C) to elicit strong inflammatory responses via TLRs and RLRs, respectively [23], [26]. Surprisingly enough, neither LPS nor polyI:C treatment could cause alteration in the expression level of the *sigmar1* gene (Figure 1C) and protein (Figure 1D) in moDCs. Statistical analysis of four independent experiments performed with cells of different blood donors revealed no significant differences in sigmar-1 protein expression following 24 h LPS or polyI:C activation as compared to the non-treated control cells indicating no direct respose to these stimuli (Figure 1E).

### NN-DMT and 5-MeO-DMT treatment inhibits inflammatory but boosts anti-inflammatory responses in LPS or polyI:C-stimulated moDCs

**Figure 2:**
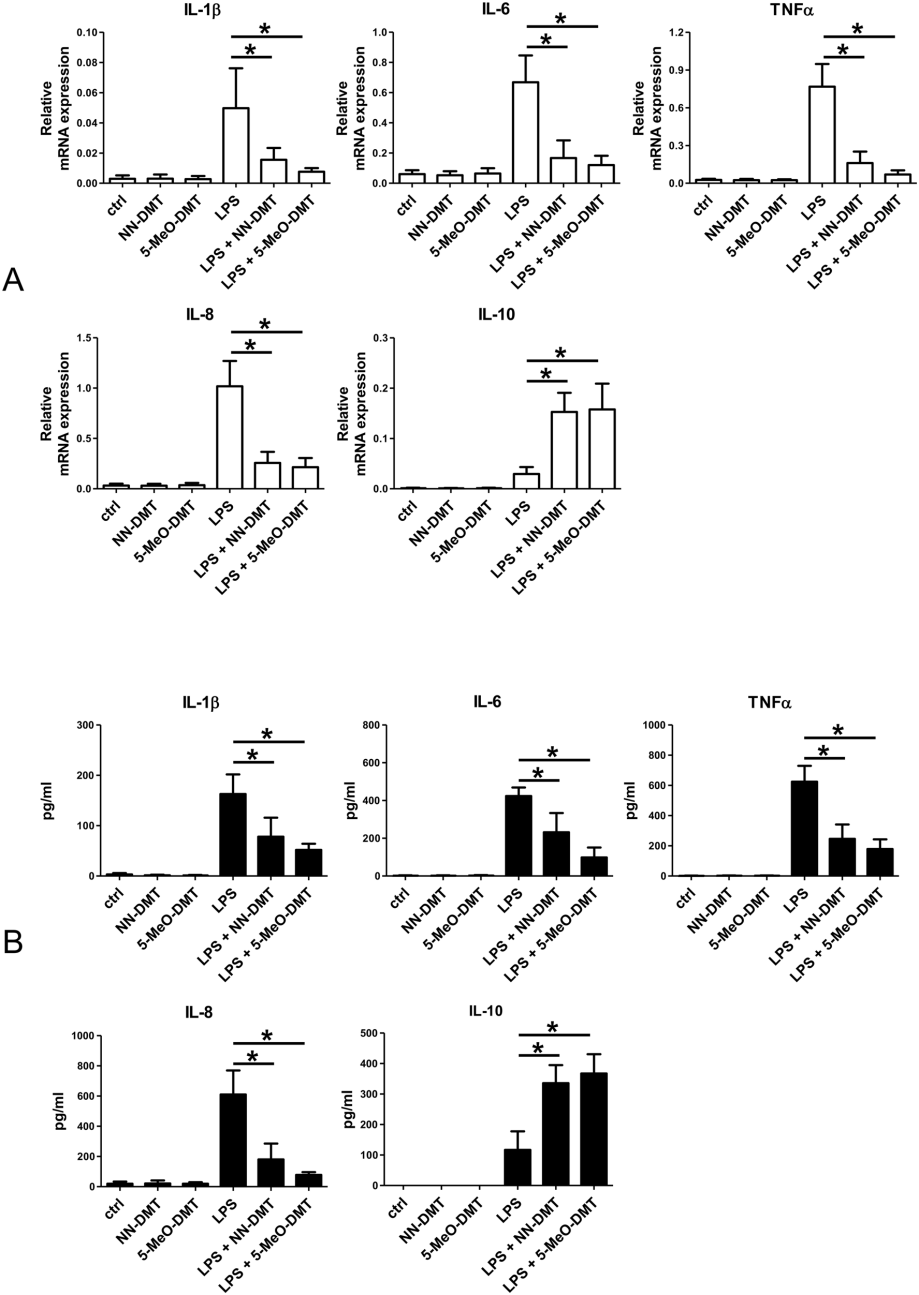
Effects of NN-DMT and 5-MeO-DMT treatment on the gene expression and secretion of cytokines and chemokines in LPS-stimulated moDCs. **A:** MoDCs were incubated with 100 µM NN-DMT (NN-DMT) or 5-MeO-DMT (5-MeO-DMT) for 8 h or left untreated (ctrl) were used as controls. Additionally, cells were either activated with 500 ng/ml LPS alone for 8 h, or pre-treated with tryptamines for 1 hour and subsequently were activated with LPS for 8 hours (LPS+NN-DMT; LPS+5-MeO-DMT). The expression of IL-1β, TNFα, IL-6, IL8, and IL-10 was assessed by real-time Q-PCR and shown as relative mRNA expression. Results represent the Mean ± SEM of 4 independent experiments. **B:** Cytokine profile of cells treated as above. Supernatants of DC cultures were collected after 24 h and were subjected to ELISA measurements. Concentration of the secreted cytokines and chemokines are shown as Mean ± SEM values of 4 independent donors. (*) means statistical significance as *p*<0.05.

**Figure 3:**
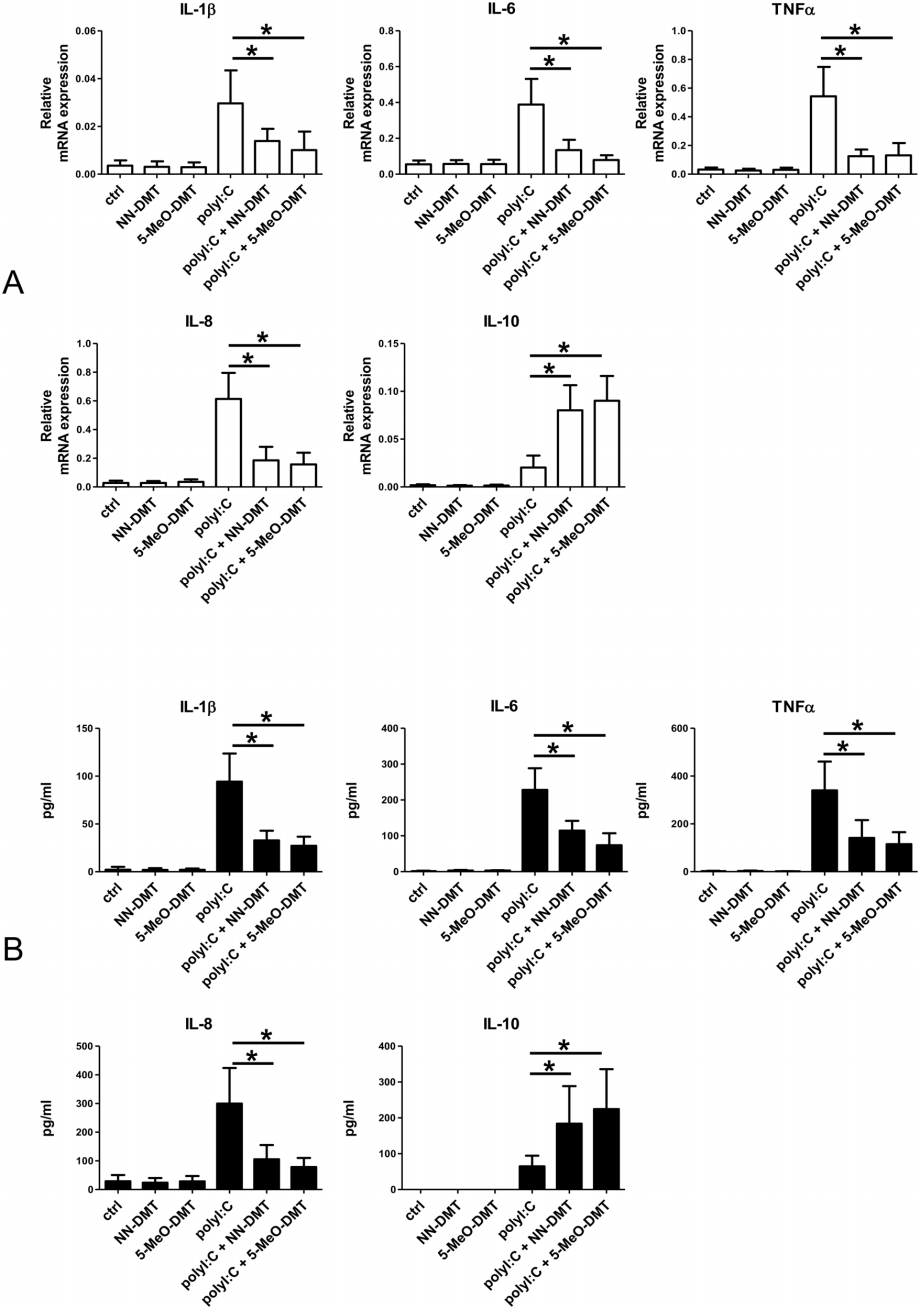
Effects of NN-DMT and 5-MeO-DMT treatment on the gene expression and secretion of cytokines and chemokines in polyI:C-activated moDCs. **A:** MoDCs were treated as in Figure 2. In this case, cell activation was induced with 20 µg/ml polyI:C for 8 h (alone, or subsequently to an 1 h 100 µM NN-DMT or 5-MeO-DMT pre-treatment). The expression of IL-1β, TNFα, IL-6, IL8, and IL-10 was assessed by real-time Q-PCR and shown as relative mRNA expression. Results represent the Mean ± SEM of 4 independent experiments. **B:** Cytokine profile of cells treated as in Fig3A. Supernatants of DC cultures were collected after 24 h and were measured by ELISA. Concentration of the secreted cytokines and chemokines are shown as Mean ± SEM values of 4 independent donors. (*) means statistical significance as *p*<0.05.

Based on the detectable levels of sigmar-1 in moDCs (Figure 1) we next tested the effects of NN-DMT and 5-MeO-DMT treatments on the cytokine profile of activated moDCs. Using different time points and concentrations of DMT (Figure S1) we were able to optimize the treatment protocol and found that a 1 h pre-treatment with NN-DMT or 5-MeO-DMT was more efficient to modulate TNF-α and IL-10 secretion than the co-administration of these agents with LPS or polyI:C (Figure S1A and S1B). This phenomenon is presumably due to the time scale required for the transportation and accumulation of DMT close to sigmar-1 sites in the cytoplasm of moDCs. We also found that 100 µM DMT, an achievable serum concentration reported by previous in vivo human [27] and animal studies [28], was optimal for modulating TNFα levels in activated moDCs and was used in further experiments (Figure S1C). Pre-treatment of LPS-activated moDC with either NN-DMT or 5-MeO-DMT strongly reduced the mRNA (Figure 2A) and the secreted levels (Figure 2B) of the pro-inflammatory cytokines IL-1β, IL-6, TNF-α, and also the chemokine IL-8/CXCL8. Decreased expression levels of these soluble regulators was consistently lower in DMT+LPS treated cells as compared to the LPS-activated control (Figure 2). Conversely, in DMT pre-treated cells the expression and production of the anti-inflammatory cytokine IL-10 was significantly higher than in LPS-treated moDCs (Figure 2A and 2B). No significant differences in the modulatory effects of NN-DMT and 5-MeO-DMT could be detected at equal working concentrations (Figure 2B), and a similar pattern of cytokine profiles of both LPS and polyI:C-stimulated moDCs was found. However, DMT pre-treated and polyI:C-activated cells had lower mRNA expression (Figure 3A) and secretion (Figure 3B) of pro-inflammatory cytokines and chemokines than the respective polyI:C-only controls. Similar to the DMT+LPS co-treatment experiments, expression and production of IL-10 was significantly higher in DMT+polyI:C treated cells than in cells treated with polyI:C alone (Figure 3). The modulation of cytokine expressions was of similar extent in cases of DMT and 5-MeO-DMT (Figure 3B). Additionally, levels of the anti-inflammatory cytokine TGFβ also elevated moderately, though not significantly, when DMT co-treatments were applied with LPS or polyI:C stimuli (data not shown). These results reflect to the strong inhibitory capacity of NN-DMT and 5-MeO-DMT on inflammatory responses in moDC when co-administered with ligands of innate immune receptors. Taken the high migratory potential and inflammatory nature of moDCs our results strongly suggest that important functional activities of these cells can be intervened by using NN-DMT and 5-MeO-DMT as pharmacological modulators.

### DMT pre-treatment of pathogen-activated human moDCs inhibits their capacity to prime autologous naive T helper 1 (Th1) and T helper 17 (Th17) cells

**Figure 4:**
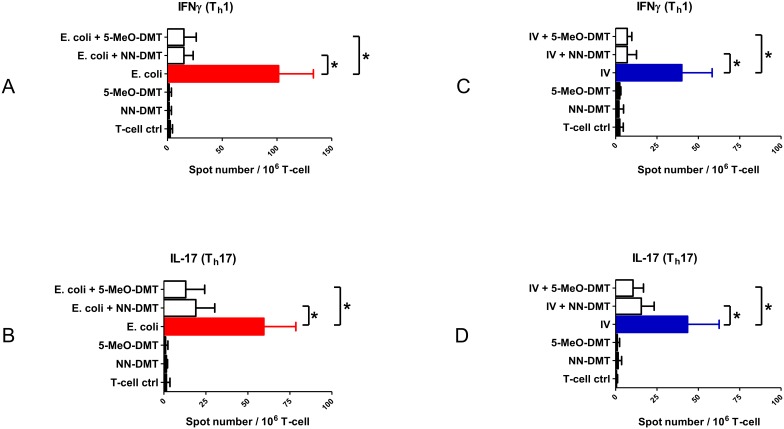
NN-DMT and 5-MeO-DMT pre-treatment of pathogen-activated human dendritic cells effectively inhibit their capacity to prime autologous naive T helper 1 and T helper 17 cells. Dendritic cells were activated either by heat-killed *E. coli* (E. coli) or inactivated influenza virus (IV) for 24 h, washed, and then co-cultured with naive autologous CD4^+^ T lymphocytes for 4 days. The number of primed, IFNγ or IL-17 secreting T cells was assessed by ELISPOT assay. T cells alone (T-cell ctrl), and non-activated DCs treated with DMT and co-cultured with autologous naive CD4^+^ T lymphocytes (NN-DMT, 5-MeO-DMT) were used as controls. **A–B:** Induction of IFNγ (A) or IL-17 (B) production of autologous naive CD4^+^ T cells induced by moDC loaded by *E. coli*. Bacteria alone (E. coli; red bars) or bacteria in combination with 1 h DMT pre-treatment (E. coli + NN-DMT, E. coli+5-MeO-DMT; empty bars) were used to activate moDCs as written above. **C–D:** Cells were activated as in Fig4A–B; in this case inactivated influenza virus (IV) was added to the moDCs alone for 24 h (blue bars), or in combination with an 1 h NN-DMT or 5-MeO-DMT pre-treatment (IV + NN-DMT, IV + 5-MeO-DMT; empty bars). Data represent Mean + SEM values of triplicate measurements of three independent donors. Asterisk indicates statistical significance (*p*<0.05).

Our results showed that short-term DMT pre-treatment of LPS or polyI:C activated moDCs are able to suppress innate pro-inflammatory responses but concommitantly promote IL-10 secretion (Figures 2 and 3). Next we sought to investigate the functional consequences of these cellular responses upon pathogenic challenge by testing moDC-mediated T-cell polarization. Previous reports showed that ligand specific stimulation of sigmar-1 is able to increase LPS-induced serum IL-10 levels while inhibit IFNγ-dependent graft-versus-host response in mice [10], [11]. It is well known that polarization of adaptive „inflammatory” Th1 response is induced and supported by IFNγ, while it is directly inhibited by IL-10, a typical effector molecule of regulatory T-cells. Th17, another inflammatory CD4^+^ helper T-cell type, is known to strongly promote inflammation and recruitment of other inflammatory cell types. The essential role of Th1 and Th17 cells in the development of inflammatory conditions in both the central nervous system and peripheral tissues in the context of infection and autoimmunity has been studied in various animal models and also in clinical studies [29]–[31]. We presumed that the opposing effects of upregulated IL-10 and inhibited secretion of inflammatory cytokines achieved by DMT administration might result in an unique T cell response. To test this hypothesis, we co-cultured naive autologous CD4^+^ T cells with pathogen-activated moDCs, and measured the number of activated IFNγ (Th1) and IL-17 (Th17) secreting effector T cells by ELISPOT assays (Figure 4). In this experimental setting the common infectious agents influenza virus and *Escherichia coli* (E. coli), both known to cause severe CNS inflammations, were used as provoking agents [32]–[34]. Activation of moDCs with heat-killed E. coli resulted in strong Th1 (Figure 4A) and Th17 (Figure 4B) responses, but both effector T cell read-outs were markedly affected when NN-DMT or 5-MeO-DMT pre-treatments were applied. The Th1 and Th17 priming capacity of moDCs in the presence of NN-DMT or 5-MeO-DMT was significantly lower than in the abscence of these drugs (Figure 4A and 4B). Similar results were obtained when the stimulated moDCs were challenged with inactivated influenza virus. DMT pre-treated and influenza-loaded moDCs were able to activate a significantly lower number of Th1 (Figure 4C) and Th17 cells (Figure 4D) as compared to influenza-only treated moDCs. These findings show that NN-DMT and 5-MeO-DMT can potently inhibit the processing and/or presentation of bacterial and viral peptide antigens by moDCs, and thus block Th1 and Th17 responses.

### NN-DMT and 5-MeO-DMT modulate the cytokine production and T cell-priming capacity of moDCs in a sigma-1 receptor dependent manner

**Figure 5:**
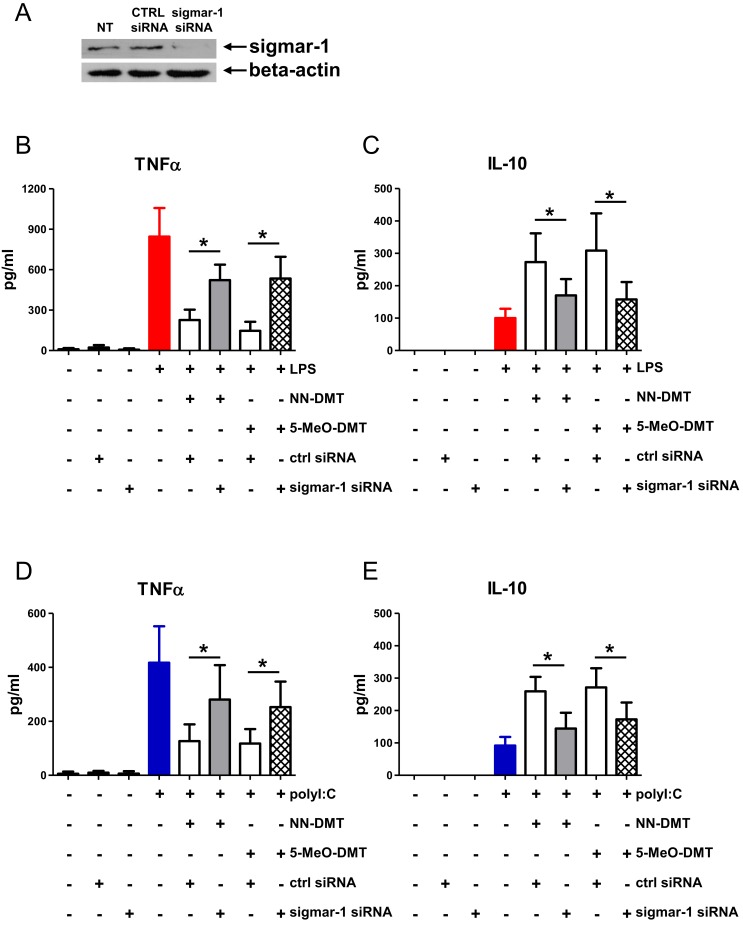
Effect of sigmar-1 gene silencing on the DMT-modified cytokine profile of LPS or polyI:C-activated moDCs. **A:** Validation of siRNA knockdown by Western blot. MoDCs were transfected with negative control siRNA (ctrl siRNA) or with gene-targeting siRNA (sigmar-1 siRNA), or left untreated (non-transfected, NT). **B–C:** Non-treated, 24 h ctrl siRNA-only, and 24 h targeting siRNA-only treated cells were used as negative controls (black bars). Red bars represent 24 h 500 ng/ml LPS-treated cells, while white bars show ctrl siRNA and 1 h DMT pre-treated cells activated with LPS for one day. Grey (NN-DMT) and checkered bars (5-MeO-DMT) demonstrate 1 h DMT pre-treated and then 24 h LPS activated sigmar-1 knockdown cells. **D–E:** MoDCs were treated as in Fig5B–C. Here, cell activation was performed with a 24 h 20 µg/ml polyI:C treatment. Blue bars represent polyI:C-only stimulation as positive control. Results are shown as Mean ± SEM of three independent donors. (*) represents *p* values <0.05. Differences are significant (*p*<0.05) in all cases of specific activation (LPS or polyI:C) versus control cells (no treatment, ctrl siRNA, sigmar-1 siRNA).

**Figure 6:**
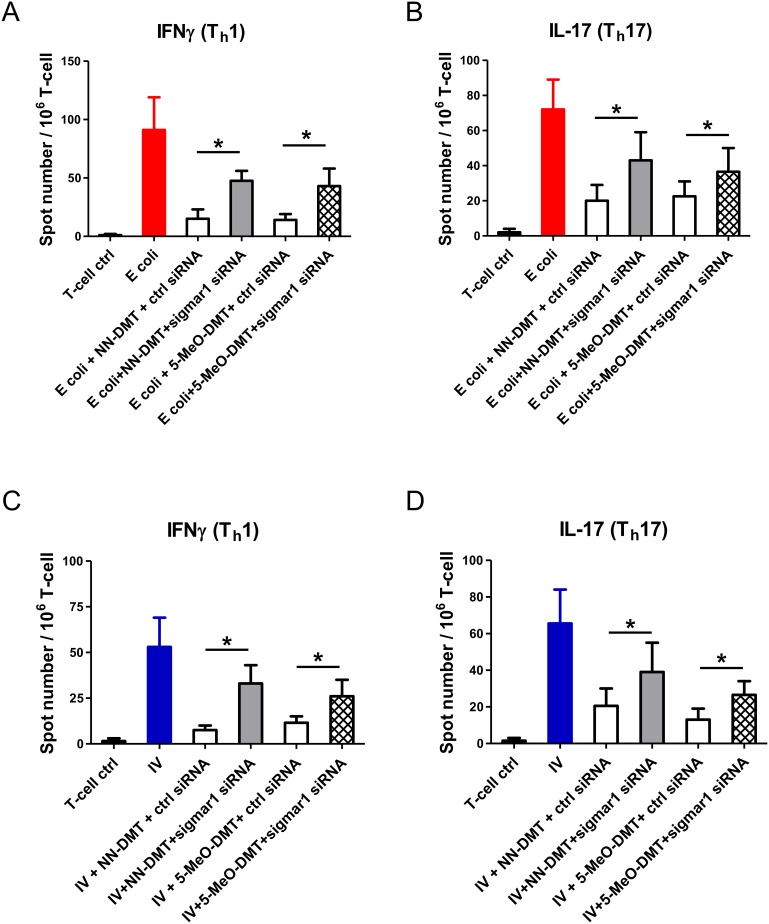
Sigmar-1 gene knockdown abrogates the inhibitory effect of tryptamines on the Th1/Th17 cell-activating capacity of pathogen-activated moDCs. Cells were co-cultured and activated, and the number of IFNγ or IL-17 secreting T cells was assessed by ELISPOT as in Figure 4. **A–B:** Red bars represent *E. coli*-loaded DCs co-cultured with autologous naive CD4^+^ T cells. White bars show ctrl siRNA and 1 h tryptamine pre-treated cells prior to a 24 h *E. coli* activation and subsequent T cell co-culturing. Grey (NN-DMT) and checkered bars (5-MeO-DMT) demonstrate 1 h tryptamine pre-treated and then 24 h *E. coli* activated sigmar-1 knockdown cells subsequently co-cultured with T cells as in Materials and Methods. **C–D:** The same setup was applied as in Fig6A–B, however, in this case inactivated influenza virus (IV) was added to moDCs as antigen stimulation. Blue bars show moDCs incubated with influenza-only for 24 h and then co-cultured with T cells (positive control). Data represent Mean + SEM values of triplicate measurements of three independent donors. Asterisk shows statistical significance (*p*<0.05). Differences are significant (*p*<0.05) in cases of specific activation (E. coli or IV) versus T-cell controls.

After demonstrating the inhibitory effect of NN-DMT and 5-MeO-DMT on the inflammatory cytokine production (Figures 2 and 3) and CD4^+^ helper T cell-priming capacity of moDCs induced by various inflammatory stimuli (Figure 4), we aimed to test whether sigmar-1 plays a role in these processes. NN-DMT has been described as a natural endogenous ligand for sigmar-1, and its closely related naturally occuring analogues, such as bufotenine or 5-MeO-DMT have also been proposed as possible sigmar-1 agonists [16]. To clarify the immunomodulatory role of sigmar-1 in moDC functional activities and to check its contribution to the observed immunomodulatory effects we performed *sigmar1* gene knock-down experiments. Specific silencing of *sigmar1* gene expression resulted in a >95% (±4%, n = 3) of downregulation of the sigmar-1 protein as compared to non-transfected and scrambled siRNA controls (Figure 5A). Using the same cell activation protocols as in Figures 2 and 3, we found that specific silencing of sigmar-1 ablated the modulatory potential of NN-DMT and 5-MeO-DMT on TNFα (Figure 5B) and IL-10 secretion (Figure 5C) by moDCs upon LPS activation. In a similar experimental setup, sigmar-1 knock-down in polyI:C activated moDCs almost completely abrogated the modulatory potential of NN-DMT and 5-MeO-DMT on TNF-α (Figure 5D) and IL-10 cytokine production (Figure 5E). We also tested the effects of sigmar-1 gene expression silencing on moDC-mediated helper T cell responses and found that downregulation of sigmar-1 resulted in significantly less inhibition of E. coli mediated Th1 (Figure 6A) and Th17 responses (Figure 6B) by dimethyltryptamines than in controls. Similarly, siRNA-based silencing of sigmar-1 expression was associated with decreased modulatory capacity of NN-DMT and 5-MeO-DMT on priming Th1 (Figure 6C) and Th17 T-lymphocyte differentiation (Figure 6D) when co-cultured with virus-loaded moDCs. Interestingly, knock-down of sigmar-1 combined with DMT pre-treatment did not restore cytokine levels and the T cell priming capacity of moDCs completely (Figures 5 and 6) raising the possibility, that other receptors and/or mechanisms may also contribute to the moDC modulatory function of dimethyltryptamines. To further confirm the involvement of sigmar-1 in this regulatory process, we used another activation model in which we pretreated moDCs with a selective and high affinity agonist of sigmar-1, i.e. PRE-084 hydrochloride, as a substitute of NN-DMT and 5-MeO-DMT. The results revealed that specific ligation of sigmar-1 with PRE-084 combined with LPS or polyI:C stimulation could by itself modulate cytokine responses of human primary moDCs (Figure S2) activated by LPS (Figure S2A and S2B) or polyI:C (Figure S2C and S2D). PRE-084 pre-treatment also resulted in significant inhibition of TNF-α production and increased IL-10 secretion that could completely be abrogated by sigmar-1 silencing (Figure S2).

## Discussion

Hallucinogenic trypamines are members of the indole alkaloid family, the largest and most common class of alkaloids in the Animal and Plant Kingdoms. NN-DMT and bufotenine, the metabolic product of 5-MeO-DMT in mammals, have been detected in animal and human blood, urine, cerebrospinal fluid, brain, intestine and many other tissues suggesting that these compounds may have important biological roles other than their psychotropic and neuromodulatory properties [12], [35]–[37].

The orphan receptor sigmar-1 has been shown to regulate many physiological processes inculding cell survival and proliferation [7], [8]. The expression of sigma receptors is not limited to the brain as high level expression was detected in mammalian liver, kidney, gut and other tissues as well [5], [38]. Sigmar-1 has also been detected in immune cells mediating strong immunosuppressive and anti-inflammatory effects [9]–[11]. It has recently been reported that NN-DMT is an endogenous ligand for sigmar-1, and its agonistic activity may be expanded to analogues, such as the methoxy derivative 5-MeO-DMT [16]. However, very little is known about the physiological functions of dimethyltryptamines in human and the emphasis of contemporary research is mostly related to understanding its psychedelic properties and to our best knowledge, the biological effects of DMT via sigmar-1 has not been investigated yet. In this study we adressed the question whether sigmar-1 is expressed in human primary myeloid cells, and if so, what is its functional role in human physiology. According to our results, sigmar-1 is expressed in human monocytes and its expression is increasing during the differentiation process to macrophages and dendritic cells (Figure 1). Although sigmar-1 expression has been documented in murine macrophages [24], [25], this is the first report on characterizing sigmar-1 expression in resting and activated human myeloid immune cells. To test the hypothesis that dimethyltryptamines may have impact on immune cell functions through sigmar-1, we tested the effects of DMT treatment on the cytokine profile of activated moDCs. In these experiments we used the TLR3/RLR ligand polyI:C and the TLR4 agonist LPS as strong inducers of innate immune responses [18]. Our results revealed that NN-DMT and 5-MeO-DMT pre-treatment potently inhibited pro-inflammatory cytokine and chemokine (IL-1β, TNFα, IL-6, IL8) expression in human moDCs stimulated by specific PRR ligands, while had opposing effect on the mRNA and protein expression of the anti-inflammatory cytokine IL-10 (Figures 2 and 3). Furthermore, NN-DMT and 5-MeO-DMT interfered with the activation and polarization of naive T-lymphocytes toward Th1 and Th17 effector T cells when co-cultured with E. coli or influenza-virus loaded human moDCs (Figure 4). These results are in good agreement with reports showing that sigmar-1 activation results in elevated IL-10, decreased IFNγ and GM-CSF levels, and inhibition of lymphocyte proliferation in mice [9]–[11]. The results also demonstrated for the fist time that NN-DMT and 5-MeO-DMT have the capability to inhibit the polarization of human moDC-primed CD4^+^ T helper cells towards inflammatory Th1 and Th17 effector lymphocytes in infectious/inflammatory settings. This is of particular importance, since Th1 and Th17 cells and the cytokines they secrete are key players in the etiology and symptomatology of many chronic inflammatory and autoimmune diseases of the CNS and other tissues [29], [30], [39]. Moreover, the mobilization of innate immune mechanisms is also well established in many psychiatric and neurological disorders [39]. In neuropsychiatric research it is an increasingly accepted hypothesis that a number of diseases affecting large populations, such as Alzheimer’s, Parkinson’s disease, Major depression are caused by chronic inflammation of the central nervous system. High-resolution whole genome-wide association studies found significant correlations between gene polymorphisms of innate immune receptors and the frequency of late onset Alzheimer’s disease (AD) [40], [41]. It has also been demonstrated in mice that the ligand specific activation of the mother’s TLRs and RLRs by LPS and polyI:C results in decreased neurogenesis, cognitive deficits, and a marked increase in the appearance and deposition of Aβ aggregates in the brain of the offspring [42], [43]. Since blood-derived monocytes were shown to be able to translocate to the CNS [22], our results could expand the role of moDCs to a more global context by suggesting their regulatory role under autoimmune or infectious inflammatory conditions in the brain.

In order to verify the contribution of sigmar-1 to the observed immunomodulatory effects we used the approach of gene-specific silencing. The results clearly demonstrated that downregulation of sigmar-1 abrogated the immunomodulatory effects of both NN-DMT and 5-MeO-DMT on cytokine secretion by innate immune cells (Figures 5) and also inhibited the moDC-mediated polarization of Th1 and Th17 effector cells (Figure 6). Remarkably, knock-down of sigmar-1 upon DMT pre-treatments could not restore cytokine levels and the T cell priming capacity of moDCs completely (Figures 5 and 6). These findings suggest that additional mechanisms through which DMT could modulate moDC functions may exist. NN-DMT and 5-MeO-DMT also bind to the 5-HT_2_ (particularly 5-HT_2A_) and 5-HT_1A_ serotonin receptors with high affinity [44], [45]. This agonism was suggested to take part in psychological effects of dimethyltryptamines but may also contribute to immunological functions, since the neurotransmitter serotonin also exerts anti-inflammatory and immunoregulatory effects in DCs [46], [47]. As the experiments with human cells were performed in serum-free medium, the „serotonin background” effect can be excluded [48]. Thus, it is very likely that the observed phenomenon is the result of DMT-mediated serotonin receptor activation.

To further verify the role of sigmar-1 in the modulation of moDC functions, we used the highly selective, high-affinity sigmar-1 agonist PRE-084 hydrochloride as a substitute of NN-DMT/5-MeO-DMT in the activation protocol. Similarly to NN-DMT and 5-MeO-DMT, PRE-084 treatment strongly interfered with TNF-α and IL-10 secretion by LPS or polyI:C stimulated moDCs (Figure S2). However, sigmar-1 knock-down could completely restore cytokine levels in PRR-activated and PRE-084-treated cells showing that sigmar-1 plays an essential role in the immunomodulation of moDCs (Figure S2).

We conclude that the function of dimethyltryptamines may extend the central nervous system activity and may play a more universal role in immune regulation. Here we demonstrate for the first time that NN-DMT and 5-MeO-DMT have potent immunomodulatory effects on the functional activities of human dendritic cells operating through the sigma-1 receptor. We also show that DMT-mediated sigmar-1 activation can interfere with both innate and adaptive immune responses. On the one hand, it strongly decreases the levels of pro-inflammatory cytokines and chemokines such as IL-1β, IL-6, TNFα and IL8, while upregulates the production of the anti-inflammatory cytokine IL-10. On the other hand, NN-DMT and 5-MeO-DMT pre-treatment of pathogen-activated moDCs abolishes their capacity to initiate adaptive immune responses mediated by inflammatory Th1 and Th17 cells. These findings greatly expand the biological role of dimethyltryptamines, which may act not only as neuromodulators or psychedelics, but also as important regulators of both innate and adaptive immunity. Thus, the DMT-sigmar-1 axis emerges as a promising candidate for novel pharmacotherapies of chronic inflammatory and autoimmune diseases.

## Supporting Information

Figure S1 **Time-dependence of the effects of sigmar-1 stimulation on moDC cytokine expression profiles. A-B:** Expression of TNFα and IL-10 genes in 8 h 500 ng/ml LPS-stimulated moDCs. Tryptamines were added to the cells either at the time of LPS treatment (co-administration; co-adm) or 1 h prior to activation with LPS (1 h pre-treatment). Red bars represent LPS-only treated positive controls. White bars demonstrate co-treatments with 100 µM NN-DMT and 500 ng/ml LPS, while black bars show co-treatments with same concentrations of 5-MeO-DMT and LPS. Results are demonstrated as Mean ± SEM of three independent donors. **C:** Concentration-dependence of tryptamines in hindering TNFα production of 24 h 500 ng/ml LPS (red line) or 20 µg/ml polyI:C (blue line)-treated moDCs. Non-activated controls are shown in green. Data of a representative experiment out of two are shown. (*) represents *p* values<0.05.
(RAR)

Figure S2 **Results of sigmar-1 gene silencing on the PRE-084 hydrochloride-modulated cytokine profile of moDCs activated by LPS or polyI:C.** Non-treated, 24 h ctrl siRNA-only, and 24 h targeting siRNA-only treated cells were used as negative controls (black bars). Red and blue bars represent 24 h 500 ng/ml LPS (red) or 20 µg/ml polyI:C (blue)-treated cells, while white bars show ctrl siRNA and 100 µM PRE-084 hydrochloride-treated cells 1 h prior to activation with LPS (**A–B**) or polyI:C (**C–D**) for one day. Green bars demonstrate 1 h PRE-084 hydrochloride pre-treated and then 24 h LPS (**A–B**) or polyI:C (**C–D**) activated sigmar-1 knockdown cells. Results are shown as Mean ± SEM of three independent donors. (*) represents *p* values<0.05.
(RAR)

Table S1 **QPCR assay information.**
(RAR)
